# Use of Generative AI for Substance-Related Information Among Young Adults (Aged 18-29 Years) in the United States: Cross-Sectional Survey

**DOI:** 10.2196/100667

**Published:** 2026-10-19

**Authors:** Mireia Triguero Roura, Pia M Mauro

**Affiliations:** 1 Center for Pharmacoepidemiology, Rutgers, The State University of New Jersey, 112 Patterson Street, New Brunswick, NJ, 08901, United States, 1 (848) 932-8413; 2 Department of Biostatistics and Epidemiology, Rutgers School of Public Health, Piscataway, NJ, United States

**Keywords:** genAI, generative AI, artificial intelligence, chatbots, substance use, young adults

## Abstract

One in three young adults reported substance-related generative AI use, mainly for seeking information on substances, with higher prevalence among Black and male respondents; findings highlight that generative AI might be shaping the substance-use ecosystem among young adults and further research is needed to understand its public health implications.

## Introduction

Since 2023, user-facing large language models (LLMs), often referred to as generative AI (genAI), have become ubiquitous, especially among young adults aged 18‐29 years [1]. As LLMs become embedded in information-seeking behavior, they may play a role in substance use as youth are more likely to use the internet to find health-related information [2]. Substance use also peaks in young adulthood, making them a high-risk group for substance use–related harms [3]. From 2018 to 2024, drugs or alcohol accounted for 25% of young adult mortality [4]. Substance-related genAI use is concerning; for instance, news reports documented the overdose death of a young adult who used genAI to learn about substance use [5].

While one in six young people use genAI for mental health [6], the prevalence of substance-related genAI use remains unknown. We aimed to (1) estimate the prevalence of substance-related genAI use among young adults, (2) identify demographic correlates of substance-related genAI use, (3) characterize reasons and frequency for use, and (4) examine perceptions of substance-related genAI use.

## Methods

### Ethical Considerations

This cross-sectional study was approved by the Rutgers Institutional Review Board (protocol: 2026000488). Informed consent was obtained online and data were collected anonymously. Participants received US $0.50 compensation.

### Sample and Design

Participants were recruited in April 2026 via Prolific, which applies participant verification, monitoring, and minimum compensation standards to yield high-quality online panel data [7]. Eligibility criteria were US residents aged 18‐29 years with a Prolific rating >97%. Quotas ensured equal representation by age (18-23 years, 24-29 years), sex (male, female), and race reflecting the American Community Survey distribution of young adults.

The survey assessed ever having used genAI, followed by substance-related genAI. Informed by prior research [6], survey items measured general and substance-related specific frequency of use (never/hardly ever/once per month/once per week/daily or almost daily). Perceived trust, reliability, and helpfulness of genAI (general and substance-related) were measured on a 5-point Likert scale (strongly disagree to strongly agree). Respondents selected each of 11 reasons for substance-related genAI use (including “other”) that applied to them, grouped into substance-specific information (eg, dosage), consequences (eg, risks), and substance use disorder (SUD)–related topics (eg, addiction).

Target recruitment was 1000 participants to achieve ±3% precision (95% confidence) in overall prevalence. The final sample (N=995) excluded 5 respondents who failed quality checks. Twenty-seven reported having never used genAI and were not asked about substance-related use; we coded them as 0 on the substance-related genAI use question. Two participants did not report sex or race, and 75 did not report student status; missing data were treated as a separate category. For methodological details, see Multimedia Appendix 1.

### Data Analysis

For this study, we (1) estimated the prevalence of substance-related genAI use; (2) examined demographic correlates with *χ*^2^ tests and a single multivariable logistic regression estimating adjusted odds ratios (aORs) of substance-related genAI use for each demographic variable while controlling for the others; (3) described reasons and frequency for substance-related genAI use; and (4) compared perceived helpfulness, reliability, and trustworthiness between general and substance-related genAI use using 2-sided paired *t* tests. Analyses were conducted in R (version 4.6.1; R Foundation for Statistical Computing).

## Results

Overall, 97.3% (968/995) reported any prior use of genAI, and 32.3% (95% CI 29.3%‐35.2%; 321/995) reported substance-related genAI use. In comparison, 57.5% (95% CI 54.4%‐60.6%; 572/995) reported substance-related Google searches (92.8% among those who reported substance-related genAI use; 298/321).

Participants who reported substance-related genAI use differed by race and sex compared with those who did not (Table 1). Black (compared to white; aOR 2.52, 95% CI 1.72‐3.7) and male (compared to female; aOR 2.04, 95% CI 1.55‐2.7) respondents were more likely to report substance-related genAI use, controlling for age and student status.

**Table 1.** Characteristics of young adults in the survey sample by substance-related genAI use.

Characteristic	Full sample (N=995), n (within-category %)	Substance-related genAI^a^ use		*χ*^2^ (*df*); *P* value^b^	Adjusted odds of any substance-related genAI use
		No^c^ (n=674), n (within-category %)	Yes (n=321), n (within-category %)		Adjusted odds ratio (95% CI)^d^ (N=995)
Racial group^e^				27.3 (5); *P*<.001	
Asian	60 (6)	43 (6.4)	17 (5.3)		0.91 (0.49‐1.62)
Black	139 (14)	68 (10.1)	71 (22.1)		2.52 (1.72‐3.7)*
Mixed	101 (10.2)	72 (10.7)	29 (9)		0.94 (0.58‐1.5)
Other	86 (8.6)	63 (9.3)	23 (7.2)		0.84 (0.49‐1.4)
White	607 (61)	426 (63.2)	181 (56.4)		(reference)
Unknown	2 (0.2)	2 (0.3)	0 (0)		—^f^
Sex				26.7 (2); *P*<.001	
Female	496 (49.8)	373 (55.3)	123 (38.3)		(reference)
Male	497 (49.8)	299 (44.4)	198 (61.7)		2.04 (1.55‐2.7)*
Unknown	2 (0.2)	2 (0.3)	0 (0)		—^f^
Age group (years)				0.5 (1); *P*=.49	
18‐23	501 (50.4)	345 (51.2)	156 (48.6)		(reference)
24‐29	494 (49.6)	329 (48.8)	165 (51.4)		1.21 (0.89‐1.63)
Student status				1.1 (2); *P*=.57	
Yes	372 (37.4)	246 (36.5)	126 (39.3)		1.22 (0.89‐1.67)
No	548 (55.1)	374 (55.5)	174 (54.2)		(reference)
Unknown	75 (7.5)	54 (8)	21 (6.5)		0.89 (0.5‐1.52)

a GenAI was described to participants as such: “Chatbots or apps that mimic human conversational language are sometimes called generative AI. Examples of generative AI include ChatGPT, Google Gemini, Meta AI, or Snap’s My AI.”

b *χ*2 test whether individual variables are associated with using genAI for substance-related information.

c Includes n=27 who said they had never used genAI for any purpose.

d Adjusted odds ratios estimated from a logistic model predicting any substance-related genAI use with all the demographic variables included in the model. * signifiies coefficients were significant at *P*<.001.

e The online survey provider (Prolific) did not include Hispanic information.

f Estimate not reported due to complete separation (all observations had outcome=0), resulting in a nonestimable odds ratio; the two “unknown” people in the sex category are the same two as in the race category.

Among those who reported substance-related genAI use, 51.1% (164/321) used it hardly ever, while 48.2% used it at least monthly (monthly=103/321, 32.1%; weekly=39/321, 12.1%; daily/almost daily=13/321, 4%). Two people reported never using it in the frequency question despite previously affirming substance-related genAI use, likely reflecting any past use but not current use. Almost all respondents who used genAI for substance-related purposes sought information on the consequences of use (92.8%, 298/321), followed by substance-specific information (156/321, 48.6%), SUD-related topics (34.9%, 112/321), and other uses (14/321, 4.4%).

Likert-scale ratings of genAI’s perceived helpfulness, reliability, and trustworthiness were high across domains (Figure 1). Perceived helpfulness ratings were higher for general use (mean 4.44, 95% CI 4.41‐4.47) than substance-related use (mean 4.12, 95% CI 4.09‐4.16). In contrast, perceived trustworthiness ratings were higher for substance-related use (mean 3.66, 95% CI 3.62‐3.71) than general use (mean 3.51, 95% CI 3.47‐3.56) and perceived reliability did not vary by domain.

**Figure 1.**
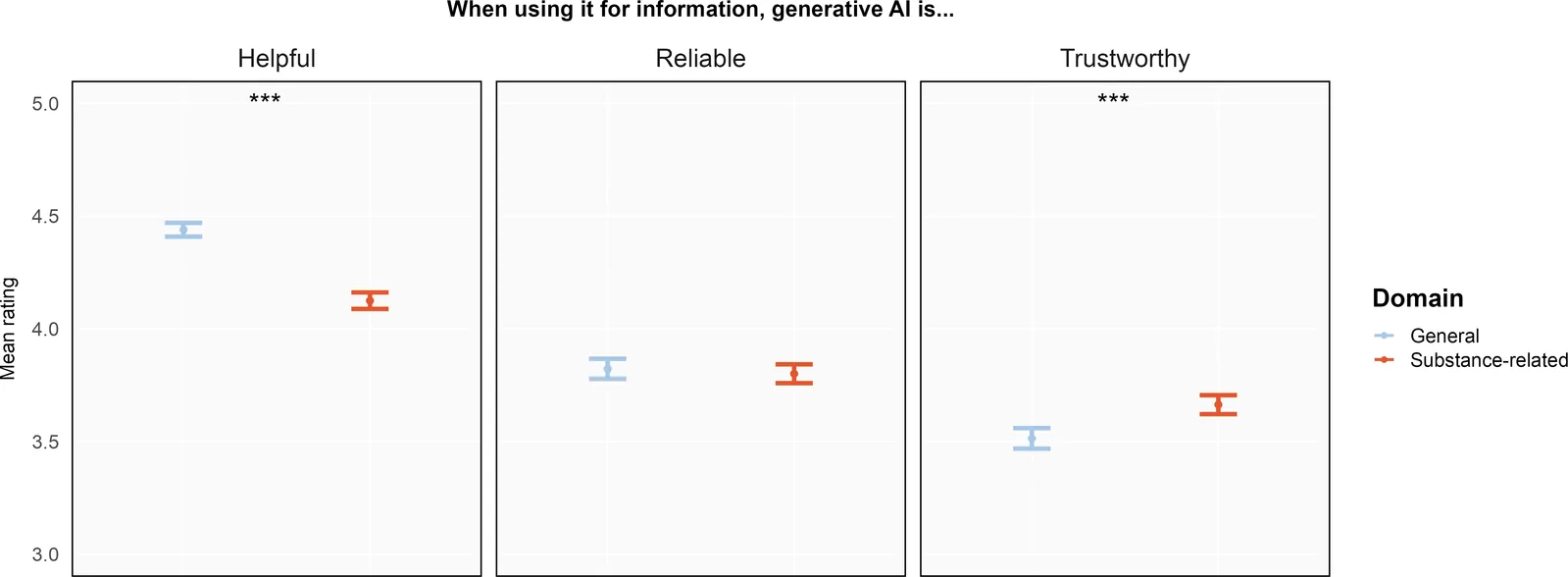
Mean ratings of perceived helpfulness, reliability, and trustworthiness of generative AI across domains. Significance stars correspond to *P* values of a 2-sided paired *t* test comparing means across domains (*P<.05; **P<.01; ***P<.001). The y-axis corresponds to a Likert scale where 1=Strongly disagree and 5=Strongly agree. Helpful scale: mean difference=−0.31, *t*_320_=−7.07, *P*<.001; reliable scale: mean difference=−0.02, *t*_320_=−0.46, *P*=.64; trustworthy scale: mean difference=0.15, *t*_320_=3.30, *P*=.001.

## Discussion

In this cross-sectional study with an online-recruited sample designed to approximate the US young adult population, one in three young adults reported substance-related genAI use; about half said they used it at least monthly for that purpose, primarily to gain information on substance-related risks and consequences. Elevated use likely reflects both broader genAI engagement and perceived benefits specific to substance-related queries, including anonymity. Black and male participants were more likely to use genAI for substance-related purposes than their counterparts, which may reflect differences in health care access, stigma, trust, and health information–seeking behaviors [8].

Young adults reported high levels of perceived trust, reliability, and helpfulness of the genAI. Findings suggest that genAI is becoming another part of the digital health ecosystems used by young adults and highlight the public health impact of information disseminated through genAI. LLMs are probabilistic language models that can easily reproduce misinformation [9], and research has shown concerning results when LLMs are tested in health contexts [10]. Studies should explore how accurate LLM-generated information is and how young adults interpret and act upon this information in substance-related contexts.

Study limitations include the use of an online, nonprobability sample, which could overrepresent highly online and genAI-aware individuals, potentially inflating genAI-related estimates and limiting generalizability. Although multiple data quality checks were implemented to exclude low-quality or automated responses, residual measurement error is possible. Measures were newly adapted for this study and were not piloted or validated; future studies should test validity.

Findings highlight how genAI might be shaping the substance-use ecosystem among young adults. Additional research is needed to understand the drivers of these demographic differences and the public health implications of substance-related genAI use.

## Supplementary material

10.2196/100667 Multimedia Appendix 1 Methodological supplement.

## Abbreviations

aOR: adjusted odds ratio
genAI: generative AI
LLM: large language model
SUD: substance use disorder
