# Supplementary material for: Use of Generative AI for Substance-Related Information Among Young Adults (Aged 18-29 Years) in the United States: Cross-Sectional Survey
Source: JMIR Form Res. 2026 Oct 19;10:e100667. doi: 10.2196/100667 (PMC13679863; doi:10.2196/100667)
Supplement: Supplementary file 1 [file formative-v10-e100667-s001.docx]

**Methodological Supplement for**

Triguero Roura, Mireia and Pia M Mauro. “Use of Artificial Intelligence for Substance Use-related information among Young Adults in the US.”

1. **Recruitment and Survey Procedure**

Participants were recruited via the Prolific platform using its stratified quota sampling tools. Eligibility criteria were restricted to U.S. residents aged 18-29 with a minimum prior approval rating of 97% on the platform. Quotas were implemented to ensure equal representation across two age bands (18–23 and 24–29) and sex (male and female). Race was proportionally sampled to approximate American Community Survey (ACS) distributions for U.S. adults aged 18–29 (White 61%, Black 14.2%, Asian 6%, Mixed 10.2%, Other 8.6%). The platform did not provide a mechanism to quota-sample based on Hispanic ethnicity. According to Prolific, samples constructed using these stratified quotas can be used to extract representative statistics of the U.S. population.^1^

The available participant pool meeting these criteria on Prolific at the time of recruitment was 40,136 individuals. We aimed to recruit 1,000 participants. Power analyses determined that with 1,000 participants, a 95% confidence interval around the prevalence estimate would have a margin of error of approximately ±3 percentage points in the most conservative case (true prevalence 50%), and smaller margins of error if the true prevalence is lower, which is the more likely scenario. A total of 1,000 participants were approved through Prolific and completed the study. An additional 21 submissions were returned (either participant voluntarily withdrew or they were screened out for not meeting age target) and 5 timed out prior to completion. Data collection occurred between April 20 and April 21, 2026. Participants received a payment of $0.50 for completion, corresponding to an effective hourly rate of approximately $24 based on a median completion time of 1 minute and 15 seconds (Q1 = 45 seconds, Q3 = 1 minute 37 seconds)

The survey was programmed and administered using Qualtrics, and all response data were stored on the Qualtrics platform. No explicit attention check items were included, as prior research suggests such checks may change user behavior, and may be more of a distraction particularly in very brief surveys.^2,3^ Instead, multiple alternative data quality safeguards were implemented. These included Prolific’s built-in bot and large language model (LLM) detection systems (the system outputs a high, mixed, or bot/LLM flag),^4^ Qualtrics CAPTCHA verification, and automated prevention of multiple submissions.^6–8^ Additionally, responses exhibiting unusually fast completion times were set to be systematically excluded as part of Prolific screening procedures.^7^ We filtered respondents with reCAPTCHA score < 0.5 and respondents with a “Mixed” or lower flag from the Prolific system. As a result, a total of 5 respondents were excluded. These 5 respondents had both a reCAPTCHA under 0.5 and a “Mixed” score in the Prolific the bot detector flag.

2. **Measures**

Following research on prevalence of “generative artificial intelligence” (genAI) use among adolescents and young adults,^9^ we developed a set of measures to assess how individuals use of genAI and specifically Large Language Models (LLMs) in the form of chatbots to obtain information related to substance use. At the outset, respondents were informed that the study aimed to examine how people use genAI tools to seek information, including on topics involving both legal and illegal substances.

Participants were then provided with a brief definition of chatbots and “genAI,” along with illustrative examples (“e.g., ChatGPT, Google Gemini, Meta AI, and Snap’s My AI)”, and asked whether they had ever used such tools. Respondents who indicated that they had not used genAI were subsequently presented with a list of reasons to characterize their non-use and asked to select all that apply. These respondents were never advanced to the questions about using genAI for substance-related purposes.

Respondents who reported having ever used genAI were asked to indicate their frequency of use. They then rated their agreement with statements assessing perceived trustworthiness, helpfulness, and reliability of genAI on a 5-point Likert scale (1 = strongly disagree to 5 = strongly agree). These respondents were then asked whether they had ever used genAI to obtain information related to substances or substance use. Examples of substances were provided (“e.g., alcohol, cannabis, cocaine, prescription opioids, ketamine, fentanyl”) to standardize interpretation. Participants who indicated that they had not used genAI for this purpose were presented with a list of predefined reasons for why not and asked to select all that apply.

Participants who reported using genAI for substance-related information were asked a parallel set of questions specific to this context, including frequency of AI use and perceived trustworthiness, helpfulness, and reliability of genAI for substance-related queries. They were also presented with a list of specific use cases and asked to select all that applied. This list was further compressed for analysis into three broad categories representing information related to “use” (items a-c), “consequences” (Items d-g) and “substance use disorder” (Items h-j). There was an “other (please specify)” (item k) that was not further classified. Finally, all respondents, regardless of their reported use of genAI, were asked whether they had ever used Google to search for information about substances or substance use to establish a baseline comparison of a much more long-established information retrieving technology.

Individual demographic characteristics were obtained through Prolific’s pre-existing participant profile data, including gender, race, age, and student status. Because age served as a primary eligibility criterion, it was also assessed independently within the Qualtrics survey; analyses reported in Table 1 rely on this directly collected measure of age. Prolific allows participants to revoke consent for sharing demographic information at any time. At the time of data collection, two participants had revoked consent to share their gender information, and 74 participants had either revoked consent or had missing/expired information for student status. These data were coded as Unknown in table 1 summary statistics. There was no other missing data.

3. **Survey Instrument:**

Q1: Chatbots or apps that mimic human conversational language are sometimes called generative AI. Examples of generative AI include ChatGPT, Google Gemini, Meta AI, or Snap's My AI.

Have you ever used generative AI?

a. Yes (Go to Q3)
b. No (Go to Q2)

Q2: You answered that you do not use generative AI, please select why not (select all that apply:

- I do not trust the information
- I do not trust the developers/owners of the technology
- I worry about my privacy
- I have ethical concerns about the technology
- Other (please specify)

Q3: Have you ever used Google or other search engines for information about substances or substance use (e.g., alcohol, cannabis, cocaine, opioid prescriptions, ketamine, fentanyl)?

a. Yes
b. No

[end of survey for respondents who answered no in Q1]

Q4: How often do you use generative AI for any purpose?

- Never
- Hardly ever
- At least once a month
- At least once a week
- Daily or almost daily

Q5-7: Based on your experience, how much do you agree with the following statements about **generative AI**? Generative AI is...

|  | Strongly disagree (1) | Somewhat disagree (2) | Neither agree nor disagree (3) | Somewhat agree (4) | Strongly agree (5) |
| --- | --- | --- | --- | --- | --- |
| trustworthy |  |  |  |  |  |
| reliable |  |  |  |  |  |
| helpful |  |  |  |  |  |

Q8: Have you ever used generative AI for information about substances or substance use (e.g., alcohol, cannabis, cocaine, opioid prescriptions, ketamine, fentanyl)?

a. Yes (Go to Q10)
b. No (Go to Q9)

Q9: You answered that you do not use generative AI for information about substances or substance use, please select why not (select all that apply):

- I do not trust the information
- I worry about potential legal consequences of using AI for this purpose
- I have all the information I need
- I am not interested in this topic
- Other (please specify)

[end of survey for respondents who answered no in Q8]

Q10: How often do you use generative AI for information about substances or substance use?

- Never
- Hardly ever
- At least once a month
- At least once a week
- Daily or almost daily

Q11-13: Based on your experience, how much do you agree with the following statements about using **generative AI** for information about **substances or substance use**? When using it for substances or substance use information, generative AI is...

|  | Strongly disagree (1) | Somewhat disagree (2) | Neither agree nor disagree (3) | Somewhat agree (4) | Strongly agree (5) |
| --- | --- | --- | --- | --- | --- |
| trustworthy |  |  |  |  |  |
| reliable |  |  |  |  |  |
| helpful |  |  |  |  |  |

Q12: You have indicated you have used generative AI to find information about substances or substance use. Which of the following best describes your usage? Please select all that apply: Find information on:

a. how much should one use (dosage)
b. how to use specific substances
c. how to obtain specific substances
d. side-effects of specific substances
e. risks related to use
f. problems related to use
g. drug interactions
h. addiction
i. addiction treatment
j. how to stop using a specific substance
k. Other (please specify)

[end of survey for respondents in this branch]

4. **Breakdown of reason to use generative for substance-related reasons:**

Table S1.

Note: This question was only asked to respondents who answered they had used genAI for substance-related purposes (n=321). Respondents were allowed to select multiple reasons for why they GenAI for substance-related-purposes. Percents are computed over the number of respondents. General reason corresponds to the broad category reported in the paper (constructed as detailed in section 2). For the specific wording of the question see section 3.

| General reason | Specific reason | Count | Percent |
| --- | --- | --- | --- |
| Substance-specific | how much should one use (dosage) | 130 | 40.5 % |
| Substance-specific | how to obtain specific substances | 23 | 7.2 % |
| Substance-specific | how to use specific substances | 68 | 21.2 % |
| Consequences of use | drug interactions | 128 | 39.9 % |
| Consequences of use | problems related to use | 171 | 53.3 % |
| Consequences of use | risks related to use | 217 | 67.6 % |
| Consequences of use | side-effects of specific substances | 233 | 72.6 % |
| SUD-related | addiction | 81 | 25.2 % |
| SUD-related | how to stop using a specific substance | 61 | 19 % |
| SUD-related | addiction treatment | 59 | 18.4 % |
| --- | Other | 14 | 4.4 % |

**References:**

1.	Prolific Research. What are representative samples on Prolific. Prolific Research. Accessed April 22, 2026. https://researcher-help.prolific.com/en/articles/445161-what-are-representative-samples-on-prolific

2.	Alvarez RM, Atkeson LR, Levin I, Li Y. Paying Attention to Inattentive Survey Respondents. *Polit Anal*. 2019;27(2):145-162.

3.	Esch DT, Mylonopoulos N, Theoharakis V. Evaluating mobile-based data collection for crowdsourcing behavioral research. *Behav Res Methods*. 2025;57(4):106. doi:10.3758/s13428-025-02618-1

4.	Prolific Research. How Prolific detects bots and AI in online research. Prolific. Accessed July 1, 2026. https://www.prolific.com/resources/how-prolific-detects-bots-and-ai-in-online-research

5.	Google. Best practices for protection from automated threats | Google Cloud Fraud Defense. Google Cloud Documentation. Accessed July 1, 2026. https://docs.cloud.google.com/recaptcha/docs/best-practices-oat

6.	Prolific Research. Understanding the Limitations of reCAPTCHA Bot Detection in Research. Prolific Research. Accessed April 22, 2026. https://researcher-help.prolific.com/en/articles/445222-understanding-the-limitations-of-recaptcha-bot-detection-in-research

7.	How to prevent and detect AI or Large Language Model (LLM) use in studies | Prolific Research. Accessed April 22, 2026. https://researcher-help.prolific.com/en/articles/445207-how-to-prevent-and-detect-ai-or-large-language-model-llm-use-in-studies

8.	Qualtrics. Fraud Detection. Qualtrics. Accessed April 22, 2026. https://www.qualtrics.com/support/survey-platform/survey-module/survey-checker/fraud-detection/

9.	McBain RK, Bozick R, Diliberti M, et al. Use of Generative AI for Mental Health Advice Among US Adolescents and Young Adults. *JAMA Netw Open*. 2025;8(11):e2542281. doi:10.1001/jamanetworkopen.2025.42281
